# Pesticides and Parkinson’s Disease—Is There a Link?

**DOI:** 10.1289/ehp.8095

**Published:** 2005-09-07

**Authors:** Terry P. Brown, Paul C. Rumsby, Alexander C. Capleton, Lesley Rushton, Leonard S. Levy

**Affiliations:** 1 Medical Research Council Institute for Environment and Health, University of Leicester, Leicester, United Kingdom; 2 National Centre for Environmental Toxicology, WRc-NSF Ltd., Medmenham, Marlow, United Kingdom

**Keywords:** epidemiology, literature review, Parkinson’s disease, pesticides, toxicology

## Abstract

Parkinson’s disease (PD) is an idiopathic disease of the nervous system characterized by progressive tremor, bradykinesia, rigidity, and postural instability. It has been postulated that exogenous toxicants, including pesticides, might be involved in the etiology of PD. In this article we present a comprehensive review of the published epidemiologic and toxicologic literature and critically evaluate whether a relationship exists between pesticide exposure and PD. From the epidemiologic literature, there does appear to be a relatively consistent relationship between pesticide exposure and PD. This relationship appears strongest for exposure to herbicides and insecticides, and after long durations of exposure. Toxicologic data suggest that paraquat and rotenone may have neurotoxic actions that potentially play a role in the development of PD, with limited data for other pesticides. However, both the epidemiology and toxicology studies were limited by methodologic weaknesses. Particular issues of current and future interest include multiple exposures (both pesticides and other exogenous toxicants), developmental exposures, and gene–environment interactions. At present, the weight of evidence is sufficient to conclude that a generic association between pesticide exposure and PD exists but is insufficient for concluding that this is a causal relationship or that such a relationship exists for any particular pesticide compound or combined pesticide and other exogenous toxicant exposure.

Parkinson’s disease (PD) is an idiopathic disease of the nervous system characterized clinically by parkinsonism: chronic progressive tremor, bradykinesia, rigidity, and postural instability. The major pathologic feature of PD is the profound loss of pigmented neurons, mainly in the pars compacta of the substantia nigra (SN). Associated with this neuronal loss is the presence of large eosinophilic inclusions, called Lewy bodies, within the remaining pigmented neurons, made up of a series of proteins, including neurofilaments, α-synuclein fibrils, ubiquitin, parkin, and proteasomal elements. The first clinical signs of PD, however, become apparent only after the loss of about 70–80% of dopaminergic neurons (Schapira 1999), and although the diagnosis of PD is entirely clinical, histopathology on autopsy is the only way to definitively confirm a diagnosis.

The mean age of onset of PD is typically between 60 and 65 years, and in Europe the prevalence of PD has been estimated to be 1.8% in persons ≥ 65 years of age (de Rijk et al. 2000), with an incidence of approximately 16–19 per 100,000 per year (Twelves et al. 2003). Although age is unequivocally associated with increasing PD risk, the underlying process of PD is distinct from the natural aging process (Goldman and Tanner 1998). PD prevalence is also similar among ethnic groups living in the same location (Morens et al. 1996), but may differ among ethnic groups living in different locations (Schoenberg et al. 1988). Genetic factors can influence the risk of PD, and higher rates of PD have been found in relatives of those with PD (Foltynie et al. 2002). However, twin studies have consistently shown low rates of concordance (5–8%) in monozygotic and dizygotic twins (Foltynie et al. 2002), suggesting that other factors play a part in the etiology of PD. The exception to this is young-onset PD (onset before 50 years of age), where concordance in monozygotic twins was 100%, suggesting a primarily genetic basis (Tanner 2003). Several specific loci have been identified that result in PD, including mutations in the *PARK 1* to *PARK 8* genes, although these forms of PD are rare and usually display atypical features of the disease (Foltynie et al. 2002).

A number of causative factors have been found to induce parkinsonism similar to that of idiopathic PD, including vascular insults to the brain, repeated head trauma, neuroleptic drugs, and manganese toxicity (Adler 1999). In particular, the toxicant 1-methyl-4-phenyl-1,2,3,6-tetrahydropyridine (MPTP) resulted in the development of acute parkinsonism, similar to the idiopathic disease in nearly all clinical, pathologic, and biochemical features, in a small group of drug addicts (Langston et al. 1983). This rekindled an interest in the possible role of exogenous toxicants in the development of PD and parkinsonism generally, in particular, compounds that are toxicologically or structurally similar to MPTP, including pesticides such as rotenone and paraquat (Goldman and Tanner 1998). Numerous epidemiologic and toxicologic studies have examined pesticides as a risk factor for PD and parkinsonism and the possible mechanisms by which pesticides may act. In addition, a number of other related risk factors have been found to be associated with the development of PD, such as farming, rural living, and consumption of well water.

To date, there has been no comprehensive literature review of the epidemiologic and toxicologic evidence to critically evaluate whether a causal relationship exists between exposure to pesticides and the development of PD or parkinsonism. In this article we summarize such a critical review, undertaken on behalf of the U.K. Advisory Committee on Pesticides.

## Methods

We conducted a search of 10 major online bibliographic databases in April 2003 to identify references pertaining to the role of pesticides in the development of PD and parkinsonism. We selected search terms used with reference to the relevant indexing terms used in Embase (Elsevier, New York, NY, USA) and MedLine (National Institutes of Health, Bethesda, MD, USA) and key words used in published literature. Supplemental literature searching methods included reviewing the reference lists of reports obtained, searching current journal issues, and consulting the Internet. Reports were included in this review if they were original reports, directly addressed the role of a pesticide or pesticides in an aspect of PD or parkinsonism, were in English or French language, and were published from 1983 onward.

## Epidemiologic Evidence

A large body of epidemiologic literature exists concerning pesticides and PD, including case reports, descriptive studies, and cohort studies, although most studies have used a case–control design. A review of case reports, case series, and incidence, prevalence, mortality, and cohort studies is available in the Supplementary Material online (http://ehp.niehs.nih.gov/docs/2005/8095/supplemental.pdf). Here we summarize the case–control studies.

We identified 40 case–control studies published since 1983; we excluded two of these (Semchuk et al. 1993; Semchuk and Love 1995) from the review because the data were included in another study (Semchuk et al. 1992) and the reports did not offer any new data. Of the remaining 38 studies, 13 were carried out in the United States, 5 in Canada, 11 in Europe, 5 in Asia, 2 in Australia, 1 in South America, and 1 was from Nigeria. Table S1 in the Supplementary Material (http://ehp.niehs.nih.gov/docs/2005/8095/supplemental.pdf) gives details of each of the studies. Three of the studies were conference abstracts ( Table S1; http://ehp.niehs.nih.gov/docs/2005/8095/supplemental.pdf), but we chose to include them in our review because they would have been peer reviewed before being accepted. The results presented in these abstracts do not differ significantly from the overall findings from other studies. In addition, we identified three autopsy studies that examined the levels of various pesticides and their metabolites in the brains of PD cases (Corrigan et al. 1998, 2000; Fleming et al. 1994). The number of cases in the studies ranged from 34 to 496, and the number of controls from 25 to 2,070, although one nested case–control study used the rest of the cohort (22,286) as their controls ( Table S1; http://ehp.niehs.nih.gov/docs/2005/8095/supplemental.pdf). The mean age of the cases ranged from < 50 years to 72 years.

**Figure 1:**
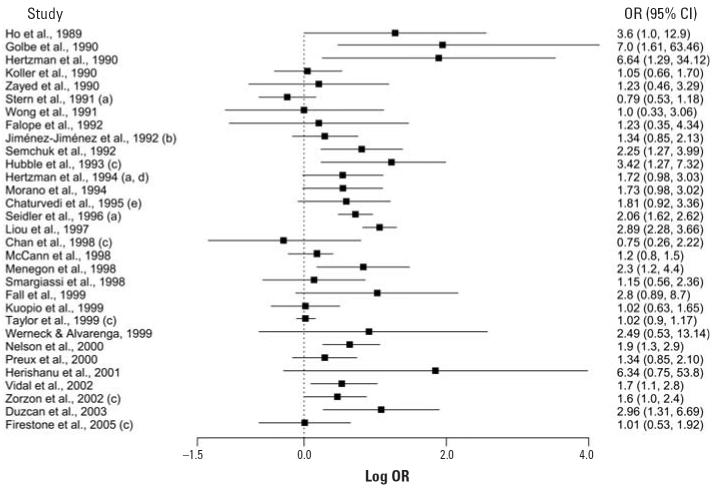
Forest plot of case–control studies examining pesticide exposure and the risk of developing PD. (**a**) Results taken from meta-analysis of Priyadarshi et al. (2000). (**b**) Unmatched calculation; figures unavailable for matched analysis. (**c**) Adjusted OR. (**d**) Assuming no missing responses and using cardiovascular patient control group. (**e**) Exposure to pesticides and fertilizers.

Figure 1 presents a forest plot of the odds ratios (ORs) for the 31 studies that presented results for exposure to pesticides as an exposure category. The ORs ranged from 0.75 to 7.0, with only two studies reporting an OR < 1.0 (Figure 1). The ORs in the remaining studies were greater than or equal to unity, of which 12 reported a significant association between pesticide exposure and the risk of PD. In the studies that found a significant relationship, the ORs ranged from 1.6 to 7.0. Confidence intervals (CIs) for a number of studies are wide, reflecting to some extent the small sample sizes. Of the seven studies not included in Figure 1, two qualitatively stated that they found no association (Kirkey et al. 2001; Tanner et al. 1989), another reported data for individual pesticide compounds only (Wechsler et al. 1991), and four reported data for individual groups of pesticides only [e.g., herbicides, insecticides (Behari et al. 2001; Butterfield et al. 1993; Gorell et al. 1998; Kamel et al. 2001)].

In a meta-analysis of 19 case–control studies published between 1989 and 1999, and using pesticides as an exposure category, Priyadarshi et al. (2000) obtained a combined OR for PD risk of 1.94 (95% CI, 1.49–2.53). The authors also analyzed the data according to geographic location and found the combined estimates were similar: United States, 2.15 (95% CI, 1.14–4.05); Canada, 1.94 (95% CI, 1.37–2.76); Europe (including one Australian study), 1.76 (95% CI, 1.41–2.21); Asia, 2.53 (95% CI, 1.58–4.05). After accounting for geographic location, heterogeneity remained between the studies. This could be due to the different inclusion/exclusion criteria used to define a case or because of the various methods used to define pesticide exposure.

**Figure 2:**
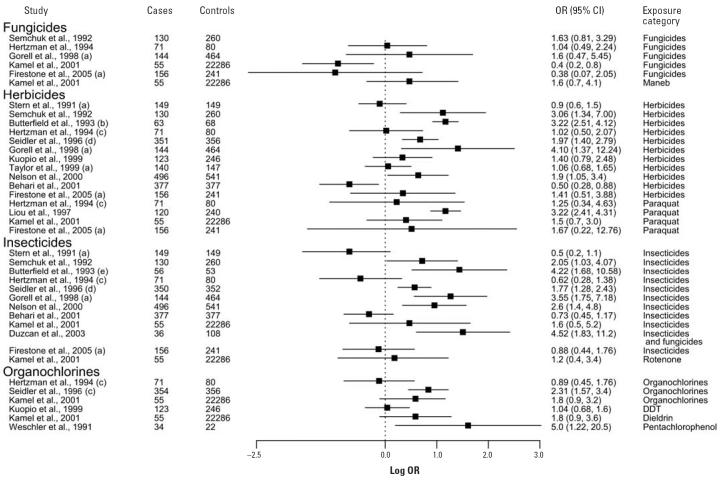
Forest plot of case—control studies looking at exposure to specific groups of pesticides or individual pesticide compounds and the risk of developing PD. (**a**) Adjusted OR. (**b**) Results taken from the meta-analysis of Priyadarshi et al. (2000). (**c**) Men only; cases compared with control group of patients with chronic cardiovascular disease. (**d**) Cases compared with regional controls. (**e**) OR recalculated from data presented.

Although most of the studies considered only pesticides or pesticides and herbicides as the exposure category, several studies attempted to examine the effects of exposure to more specific groups of pesticides (e.g., herbicides, insecticides). The ORs and 95% CIs for these studies are presented in Figure 2. In most studies, a positive association was observed between exposure to herbicides and PD risk. In one study, exposure to herbicides was a significant independent risk factor after adjustment for insecticide and other exposures (Semchuk et al. 1992). Exposure to insecticides is also generally positively associated with PD (Figure 2). Fungicide exposure was not found to be a significant risk factor for PD, nor was exposure to rodenticides (Behari et al. 2001) or acaricides (Hertzman et al. 1994; data not shown).

Several studies investigated the relationship between exposure to individual pesticides and PD risk. In two studies, paraquat exposure was shown to be significantly associated with PD (Hertzman et al. 1990; Liou et al. 1997), especially with > 20 years of exposure (Liou et al. 1997). However, other studies have not found a significant association, although PD risk was still elevated (Firestone et al. 2005; Hertzman et al. 1994; Kamel et al. 2001). Other specific groups of pesticides have also shown positive associations with PD, including organochlorines (Figure 2). Three autopsy case–control studies found increased levels of dieldrin and lindane in the brains of deceased PD patients compared with other diseased brains (Corrigan et al. 1998, 2000; Fleming et al. 1994). Positive associations were also seen with exposure to organophosphates and carbamates pesticides (Firestone et al. 2005; Wechsler et al. 1991; data not shown), although in only one study was the association significant (Seidler et al. 1996).

The relationship between exposure duration and PD risk was investigated in six case–control studies. Four found a significant association between increasing pesticide exposure duration and PD risk (Chan et al. 1998; Gorell et al. 1998; Liou et al. 1997; Seidler et al. 1996), although the relationship in one study did not remain significant after adjusting for various confounding factors (Chan et al. 1998). The remaining two studies showed nonsignificant positive associations with exposure duration (Jiménez-Jiménez et al. 1992; Zayed et al. 1990). These studies suggested that PD risk is increased when the duration of exposure to pesticides exceeds a particular threshold, because associations were often only significant for the longest exposure duration categories (e.g., > 10 or > 20 years). A positive association was also observed with high doses of pesticides compared with low doses (Nelson et al. 2000), although the risk with regular use was seen to be lower compared with occasional use (Kuopio et al. 1999). In addition, several studies observed a positive correlation with duration of exposure to, and high doses of, herbicides and insecticides (Nelson et al. 2000; Seidler et al. 1996). Significant increases in PD risk were also associated with a history of occupational use of pesticides between the ages of 26 and 35 years, herbicides between the ages of 26 and 35, 36 and 45, and 46 and 55 years, and insecticides between the ages of 46 and 55 years (Semchuk et al. 1992).

A number of potentially confounding exposures, such as well-water consumption, farming, and rural living, have also been found to be associated with an increased risk of PD in a number of studies. In a few of these studies, multivariate analyses were performed to examine the relationship between the various risk factors. Koller et al. (1990) found that well-water consumption was dependent on rural living, suggesting the risk factors were interrelated. In one study, well-water use was found to be positively and independently associated with PD (Zorzon et al. 2002), and a meta-analysis indicated the overall risk estimate to be 1.26 (95% CI, 0.96–1.64; Priyadarshi et al. 2001). Several studies have also found farming to be an independent risk factor, in addition to pesticide exposure (Gorell et al. 1998; Zorzon et al. 2002), and the meta-analysis of Priyadarshi et al. (2001) gave a combined risk estimate of 1.42 (95% CI, 1.05–1.91).

Age is unequivocally associated with increasing risk of PD and, along with sex, was used as the major matching criterion for control selection in most of the reports reviewed or was adjusted for in multivariate analyses. In the epidemiology of PD, significant associations have been reported with several other risk factors, including head trauma and family history (positive associations), and smoking and caffeine intake (negative associations). A recent meta-analysis (Hernán et al. 2002) showed that the overall risk of PD was 30% lower among coffee drinkers and 60% lower among smokers, suggesting that these factors are protective. However, although 21 of the 38 case–control studies reported ORs for the association between PD and smoking, few adjusted for it in any multivariate analysis.

### Study design issues.

Although the findings are consistent with an association between PD and pesticide exposure, there are a number of study design issues that need consideration when interpreting the results of the epidemiology studies.

#### Case ascertainment and control selection.

Many studies selected cases and controls from hospitals or clinics. Other sources of cases included lists of patients receiving anti-PD drugs, residential care centers, community or support groups, or door-to-door surveys. Sources of controls included the general population, the spouses of cases, electoral rolls, subjects suggested by their cases, and friends and relatives of the cases. Use of hospitals could result in selection bias for both cases and controls if attendance was influenced by factors such as severity of PD (with particularly severe or mild conditions being admitted elsewhere or not attending), geographic location, and social class. The use of neighborhood controls or friends and relatives of cases can result in the exposure prevalence being similar in both cases and controls, resulting in overmatching, driving the risk estimate toward the null.

#### Study size.

The size of the studies identified varied considerably. Power calculations were given in only two of the 38 case–control studies we identified, with the sample size being sufficient to detect a difference in one study (Seidler et al. 1996) but not in the other (Kirkey et al. 2001). A third study stated it had sufficient power to detect a difference in relation to pesticides as an exposure category but not in relation to subgroups of pesticides (Semchuk et al. 1992). The sample size required for an unmatched case–control study to detect an OR of 2.0 [based on Priyadarshi et al. (2000)] with a significance level of 0.05 and 90% power, assuming an estimated exposure rate (proportion exposed) among the controls of 0.10, would be slightly < 400 each for cases and controls. For ORs > 2.0, the sample size needed would be smaller, and for an increased proportion exposed among the controls, the sample size required would be larger. In matched case–control studies, the power and sample size depend on the expected number of discordant pairs (i.e., pairs in which the case and control have different exposures). Of the 38 case–control studies identified, only three had > 375 cases (Behari et al. 2001; Nelson et al. 2000; Seidler et al. 1996); the majority (31 studies) had ≤ 150 cases ( Table S1; http://ehp.niehs.nih.gov/docs/2005/8095/supplemental.pdf). The ORs for most of the studies were < 2.0 with various proportions of exposed controls, giving powers to detect an association of much less than 90%.

#### Diagnosis.

A variety of diagnostic criteria were used in the studies reviewed. A number of studies simply stated diagnosis was confirmed by a neurologist, but most defined a case on the basis of the presence of two or more of the cardinal signs of PD (tremor, rigidity, bradykinesia, and postural instability); some also used additional criteria, including responsiveness to l-dopa therapy and/or a progressive disorder. Other diagnostic criteria used included the Unified Parkinson’s Disease Rating Scale, the Hoehn and Yahr PD Staging Scale, and the UK PD Society Brain Bank Clinical Diagnosis Criteria (Fahn and Elton 1987; Hoehn and Yahr 1998; Hughes et al. 1992a) (Table S1; http://ehp.niehs.nih.gov/docs/2005/8095/supplemental.pdf).

Clinicopathologic studies have assessed the accuracy of these criteria and shown significant false-positive and false-negative rates for diagnosing PD (Hughes et al. 1992b; Litvan et al. 1998, 2003). Misdiagnosis is especially common during the early stages of the disease, even among movement disorder specialists (Litvan et al. 1996). The Movement Disorder Society Scientific Issues Committee suggested that this limitation could strongly affect the power of epidemiologic studies and clinical trials (Litvan et al. 2003) to detect a risk, by classifying individuals as cases when they should not be.

### Statistical analysis.

Multiple comparisons were carried out in all the case–control studies. Some of the observed associations may therefore have occurred by chance alone. Only a few studies adjusted PD risk from pesticide exposure for other factors or carried out multivariate logistic regression of the data. Some of the studies that did undertake multivariate analysis did not include pesticide exposure in the predictive models (Behari et al. 2001; McCann et al. 1998; Preux et al. 2000; Wong et al. 1991; Zorzon et al. 2002). A few studies found that pesticide exposure was not a significant risk factor after adjustment for confounding variables (Chan et al. 1998; Stern et al. 1991; Taylor et al. 1999; Werneck and Alvarenga 1999). In contrast, pesticide exposure was shown to be a significant risk factor after adjustment in several studies (Butterfield et al. 1993; Gorell et al. 1998; Hertzman et al. 1990; Hubble et al. 1993; Liou et al. 1997; Menegon et al. 1998; Seidler et al. 1996; Semchuk et al. 1992; Zorzon et al. 2002). These studies were not consistent in the variables used to adjust risk, and some did not include risk factors found to be associated with PD and related to pesticide exposure, such as rural living, well-water consumption, and farming as an occupation, which could result in residual confounding. Studies that have investigated these factors in relation to PD have found ORs to be generally of the same order and direction as those for pesticide exposure. Many studies have postulated that these factors and exposure to pesticides are closely linked and interrelated. However, there still remains uncertainty as to the exact nature of the relationship between farming, rural living, and pesticide exposure and their relationship to PD risk.

### Exposure assessment.

Assessment of exposure to pesticides relied upon subjects recalling their lifetime exposures over some previous 20–30 years, leading potentially to differential recall bias. For individuals occupationally exposed to pesticides, the accuracy of their historical self-reported pesticide exposure was high for broad categories of pesticides and commonly used pesticides, but not for specific pesticides (Engel et al. 2001; Hoppin et al. 2002). However, the accuracy of recall for nonoccupational or residential exposure is questionable (Teitelbaum 2002), and the authors of most studies stated that they were unable to identify specific pesticides used, owing to the subjects’ lack of knowledge about their exposures. A few studies cross-checked the answers to the administered questionnaire on a small sample of subjects and showed good test/retest reliability (Butterfield et al. 1993; Hertzman et al. 1990; Hubble et al. 1993; Koller et al. 1990; Wang et al. 1994). The questions used to assess pesticide exposure varied considerably between studies and in some reports were not given. A number of studies simply asked “Have you ever been exposed to pesticides?” (Behari et al. 2001; Kuopio et al. 1999; Preux et al. 2000; Smargiassi et al. 1998; Tanner et al. 1989; Wechsler et al. 1991; Zorzon et al. 2002), whereas others asked more detailed questions, such as “Have you sprayed pesticides or insecticides at least once a year for 5 years (not necessarily consecutively)?” (Golbe et al. 1990) and “Have you been exposed to pesticides for more than 20 days during a year for at least 10 years?” (Duzcan et al. 2003). For all these, if a positive answer was given to the question, then the participant was deemed to have been exposed to pesticides. It is thus likely that the exposures estimated from these questions will not be the same and that the estimations of PD risk for subjects classified as ever or never exposed in these studies are probably not comparable. In addition, the assessment of exposure in most studies does not take into account the timing of exposure compared with onset of symptoms, the dose of pesticide, the mechanism of exposure, or the chemical classes of the pesticides. Furthermore, the exposure category pesticides represents many hundreds of chemicals, and these may not be comparable between studies. It could be that exposure to only a few pesticide compounds results in an increased risk of developing PD; however, differences in exposure to these compounds would be masked by the use of broad pesticide exposure categories in these studies, probably contributing to the heterogeneity in risk estimates observed.

## Toxicologic Evidence

**Figure 3:**
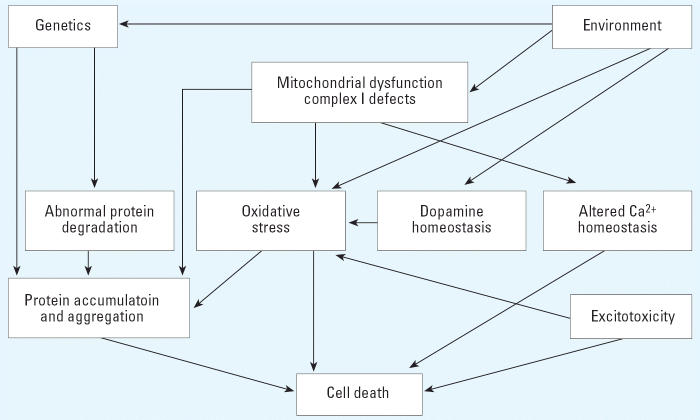
Potential mechanisms involved in the development of PD. Pathways are considered interdependent and are not necessarily mutually exclusive. Adapted from Betarbet et al. (2002).

Given the complexity of the many factors and substances to which the populations described in the epidemiologic studies have been exposed, in this section we review experimental studies on relevant pesticides to gain an insight on whether single or groups of pesticides, or related substances, may contribute to the apparent increase in PD seen in these populations. The likely underlying cellular mechanisms of PD and the involvement of exogenous factors are summarized in Figure 3. These are discussed further in the Supplementary Material (http://ehp.niehs.nih.gov/docs/2005/8095/supplemental.pdf).

There are a number of factors that should be considered when assessing the mechanistic evidence for a role for pesticides in PD development and to identify further candidate substances for consideration in experimental or epidemiologic studies: *a*) effects on the striatal dopaminergic system (these may include a decrease in dopamine levels and/or an increase in dopamine turnover as a short-term compensatory mechanism, which would be identified by an increase in metabolites or the enzyme tyrosine hydroxylase); *b*) effects on the SN (most dopaminergic neurons are present in the basal ganglia, including the SN, and changes in the SN—although not neccessarily specific—would be expected to occur with an agent involved in the development of PD); and *c*) mechanistic effects (for example, on oxidative stress, mitochondrial dysfunction/complex I inhibition, and α-synuclein levels and aggregation.

### Pesticides.

Potential mechanisms of toxicity of a number of specific pesticides are considered below.

#### Rotenone.

Rotenone is a naturally occurring insecticide and is a well-characterized, high-affinity specific inhibitor of complex I (NADH-dehydrogenase). It is extremely hydrophobic and crosses biologic membranes easily. Therefore, unlike MPTP, rotenone does not require a dopamine transporter (DAT) for access to the cytoplasm and therefore is likely to produce systemic inhibition of complex I (Betarbet et al. 2000).

Continuous infusion of rats with rotenone reduces specific complex I binding by 75%, at a low free-rotenone concentration in the brain of about 20–30 nmol/L, accompanied by nigrostriatal dopaminergic lesions, suggesting that striatal nerve endings are affected earlier and more severely by rotenone than are nigral cell bodies (Betarbet et al. 2000). Rats with these lesions had cytoplasmic inclusions containing α-synuclein in the nigral neurons, which resembled the pale body precursors to Lewy bodies found in humans with PD. Rotenone-treated animals also developed motor and postural deficits characteristic of PD, the severity of which correlated with the extent of the pathologic lesions, even after cessation of the rotenone treatment. However, Betarbet et al. (2000) also reported that rotenone seems to have little toxicity when administered orally (Sherer TB, Greenamyre JT, unpublished data).

Other experiments suggest that dopaminergic synapses in the SN pars compacta and in the nigrastriatal pathway are sensitive to the action of rotenone (Alam and Schmidt 2002). This is in contrast to the findings of Betarbet et al. (2000), who found that changes in the SN were later events. In behavioral tests, the treated animals showed a dose–dependent increase in catalepsy and decrease in locomotion. The authors surprisingly suggested that this (sub)chronic intraperitoneal dosing was comparable with chronic environmental exposure and was thus comparable with a real-life situation.

In mice and rat neuron–glial cell cultures, a nontoxic or minimally toxic concentration of rotenone and the inflammatory agent lipopolysaccharide synergistically induced dopaminergic degeneration (Gao et al. 2003). Niehaus and Lange (2003) have suggested that inflammatory factors such as lipopolysaccharide might be an environmental factor in the development of PD. The presence of brain microglia has been implicated in rotenone neurotoxicity, and these cells release reactive oxygen species as well as inflammatory factors (Gao et al. 2002; Liu and Hong 2003).

#### Paraquat.

Paraquat is a nonselective contact herbicide with high pulmonary toxicity (Corasaniti et al. 1998). One of the major considerations in relation to the potential neurotoxicity of paraquat is the extent to which it can cross the blood–brain barrier (BBB). Paraquat is a charged molecule, which may not cross the BBB, and it is not metabolized to a species more likely to gain access to the brain (Sanchez-Ramos et al. 1987). Naylor et al. (1995) found that after subcutaneous administration to neonatal, adult, and aging rats, most of the paraquat associated with structures lying outside the BBB (pineal gland and linings of the cerebral ventricles) or without a BBB [anterior portions of olfactory bulb, hypothalamus, and area postrema (Naylor et al. 1995; Widdowson et al. 1996)]. Overall, paraquat did not appear to pose a major neurotoxicologic risk in brain areas with a functional BBB. However, in the only study identified in which paraquat was given orally, neonatal mice dosed on gestation days 10 and 11 showed hypoactivity and reductions in striatal dopamine and dopamine metabolite levels (Fredriksson et al. 1993); this contrasts with the increase in activity and dopaminergic systems associated with PD-like mechanisms.

Other groups have reported that paraquat administered by intraperitoneal injection can cross an intact BBB (Corasaniti et al. 1998; Shimizu et al. 2001). Further experiments suggested the involvement of the neutral amino acid transporter in the carriage of paraquat into the brain, followed by transportation into striatal, possibly neuronal, cells, in a Na^+^-dependent manner (Shimizu et al. 2003). Inhibition of paraquat uptake into rat striatal tissues, including dopaminergic terminals, has also been shown to operate by a specific dopamine-transport inhibitor (Shimizu et al. 2001).

Although not directly relevant to human exposure pathways, paraquat has been shown to be neurotoxic after direct injection into areas of the brain (Bagetta et al. 1992; Calò et al. 1990; Corasaniti et al. 1992, 1998; De Gori et al. 1988; Iannone et al. 1988, 1991). Depending on the brain region into which the paraquat was injected, it produced different behavioral patterns, increased locomotor activity, and caused convulsions; these effects were accompanied by neuronal cell death. In general, these studies suggest that paraquat neurotoxicity is not specific to the dopaminergic nigrostriatal system because effects were observed when paraquat was injected into regions of the brain where other neurotransmitter systems are located.

Several studies have observed neurotoxicity after systemic administration of paraquat. An increase in dopaminergic neuronal death in the SN pars impacta was observed in treated rats, with no depletion in striatal dopamine but enhanced dopamine synthesis indicated by increased tyrosine hydroxylase activity (McCormack et al. 2002). The authors suggested that the apparent discrepancy between neurodegeneration and a lack of dopamine loss was probably a reflection of compensatory mechanisms by which neurons that survive damage were capable of restoring neurotransmitter tissue levels.

When rats were treated intravenously with paraquat, the brains had lower complex I activity and higher levels of lipid peroxides (indicating free radical activity) and a lower level of dopamine in the striatum (Tawara et al. 1996). Mice treated with paraquat showed an up-regulation and aggregation of α-synuclein (Manning-Bog et al. 2002). However, the studies of Woolley et al. (1989) in mice and of Naylor et al. (1995) in rats (detailed above) showed no neurotoxic effects or changes in brain dopamine levels.

#### Combination of paraquat and maneb.

Maneb [manganese ethylenebisdithiocarbamate (manganese-EBDTC)] is a dithiocarbamate herbicide, and the areas of use of maneb and paraquat have a marked geographic overlap in the United States (Thiruchelvam et al. 2000a). Mice exposed to paraquat or maneb, either alone or in combination, showed a sustained decrease in motor activity only in the combined exposure groups, with increased striatal dopamine and dopamine metabolite levels immediately postinjection, decreasing after 7 days, and reduced levels of tyrosine hydroxylase and DAT in the dorsal striatum (Thiruchelvam et al. 2000a, 2000b).

Combined exposure thus potentiated effects that appear to target the nigrostriatal dopaminergic systems. The authors suggested that mixtures of pesticides could play a role in the etiology of PD. In a series of studies on developmental exposure to the combined pesticides, mice had reduced motor activity and striatal dopamine levels (Thiruchelvam et al. 2002). Although the greatest loss of nigrostriatal dopaminergic cells was seen after combined treatment, there was significant loss with all treatments after rechallenge when adult, suggesting that a state of silent toxicity had been unmasked upon adult rechallenge. There was also evidence that prenatal exposure to maneb may lead to alterations of the nigrostriatal dopaminergic system and enhanced susceptibility to adult exposure to paraquat (Sherer et al. 2002).

In a further study on mice of different ages using higher doses (Thiruchelvam et al. 2003), reduction in locomotor activity and motor coordination and reduction in dopamine metabolites and turnover were greatest in the oldest mice (18 months of age). The decrease in the number of nigrostriatal dopaminergic neurons was progressive, particularly in the oldest mice given paraquat and maneb in combination. The result demonstrates an enhanced sensitivity of the aging dopamine pathway particularly to paraquat and maneb.

Exposure of transgenic mice to maneb and paraquat showed a higher level dopaminergic neurotoxicity in mice with doubly mutated α-synuclein compared with mice expressing normal wild-type human α-synuclein (Thiruchelvam et al. 2004).

Another report from the same group (Barlow et al. 2003) suggested that a number of different dithiocarbamates potentiate the toxicity of both MPTP and paraquat in mouse models of parkinsonism. This included the increased accumulation of dopamine in synaptosomes due to delayed efflux.

#### Dithiocarbamates.

There is some evidence for the neurotoxicity of dithiocarbamates, including studies on the manganese-containing pesticide maneb, alone or in combination with paraquat. Although manganese has been shown to cause PD-like effects in workers at high occupational exposure, it affects the globus pallidus rather than the SN and is also resistant to the beneficial effects of l-dopa. However, neurotoxic effects have been observed in toxicologic studies with the non-manganese-containing parent compound, EBDTC, from which maneb is derived (McGrew et al. 2000).

Acute exposure of mice to maneb led to central nervous system depressant effects, including decreased locomotor activity involving the dopaminergic systems (Morato et al. 1989; Takahashi et al. 1989). EBDTC (McGrew et al. 2000) and diethyldithiocarbamate (Miller et al. 1991) enhance both neurobehavioral effects and striatal dopamine depletion of MPTP in mice. *In vitro* studies on rat mesencephalic–striatal primary cocultures, using both mancozeb (manganese-zinc-EBDTC) and zineb (zinc-EBDTC), showed similar inhibitory activity on dopamine and GABA uptake for both compounds (Soleo et al. 1996). The authors suggested that EBDTC rather than manganese might be responsible for the cytotoxic effects on neuronal systems and that the findings were relevant to the pathophysiology of parkinsonism.

In a study by Zhang et al. (2003), maneb administered directly to the lateral ventricles of rats showed induction of dopaminergic neurodegeneration and extensive striatal dopamine efflux, comparable with that induced by the metabolite of MPTP, 1-methyl-4-phenylpyridine (MPP^+^).

#### Cyclodienes.

Bloomquist and colleagues have carried out studies examining possible effects of the organochlorine cyclodiene pesticides, in particular, dieldrin and heptachlor, on possible biomarkers of PD. Heptachlor increased the maximal rate of striatal dopamine uptake, which was attributed to induction of the DAT and a compensatory response to elevated synaptic levels of dopamine (Bloomquist et al. 1999; Kirby et al. 2001; Miller et al. 1999). Kirby et al. (2001) suggested that heptachlor and perhaps other organochlorine pesticides exert selective effects on striatal dopaminergic neurons and may play a role in the etiology of PD. Heptachlor and dieldrin administered to pregnant rats, although causing some maternal toxicity, resulted in increased activity of striatal DAT in offspring exposed during gestational, perinatal, and adolescent periods (Purkerson-Parker et al. 2001).

There is some evidence that dieldrin may interfere with electron transport and increase the generation of superoxide radicals (Stedeford et al. 2001). In proliferating PC12 cells exposed to dieldrin, there was evidence for increased oxidative stress. In mesencephalic cell cultures (Sanchez-Ramos et al. 1998) and PC12 cells (Kitazawa et al. 2001), there was a rapid release of dopamine and its metabolite, followed by apoptotic cell death.

Although the convulsant and proconvulsant actions of endosulfan have been attributed to an antagonistic action on GABA, a dopaminergic involvement has been suggested for its induction of hypermotor activity and circling movement (Ansari et al. 1987; Paul and Balasubramaniam 1997). Administration of endosulfan during gestation and lactation in rats up to 2–3 weeks of age produced a significant decrease in the affinity and maximum numbers of striatal dopaminergic receptors without affecting other receptor profiles, suggesting that dopaminergic receptors are unusually sensitive to the action of endosulfan (Seth et al. 1986).

#### Pyrethroids.

During investigations into the possible involvement of the pyrethroid permethrin and the organophosphate chlorpyrifos on the etiology of PD and Gulf War illness, mice treated with permethrin showed increased dopamine uptake at low doses (e.g., 134% at 1.5 mg/kg), whereas at higher doses dopamine uptake was depressed [e.g., 50% at 25 mg/kg (Karen et al. 2001)]. Reduced mitochondrial function was observed in *in vivo* synaptosome preparations, and although striatal dopamine levels were not decreased, there was an increased dopamine turnover and decreased motor activity. Although frank parkinsonism was not observed, dopaminergic neurotransmission was affected by exposure to permethrin.

Mice treated with deltamethrin showed a 70% increase in maximal dopamine uptake in *ex vivo* synaptosomes suggestive of an up-regulation in DAT expression (Kirby et al. 1999). Unlike MPTP, deltamethrin did not decrease dopamine, although there was some evidence of increased turnover.

When the pyrethroid insecticide fenvalerate was given orally to rats, there was a pronounced, but not dose-related, inhibition of dopamine and its metabolites and decreased dopamine binding in several brain regions, including the corpus striatum (Husain et al. 1991). In another study, fenvalerate or cypermethrin given during gestation and lactation to pregnant and nursing dams (Malaviya et al. 1993) showed a significant increase in dopamine and muscarinic receptors of striatal membranes in the pups. Malaviya et al. (1993) suggested that the findings demonstrated disturbance of both the dopaminergic and cholinergic pathways.

#### Other pesticides.

Although there is evidence for neurotoxic effects of some other pesticides, all the mechanistic systems seen in PD are not consistently effected. A review of the potential involvement of these other pesticides is presented in the Supplementary Material (http://ehp.niehs.nih.gov/docs/2005/8095/supplemental.pdf).

### Interaction of pesticides with α-synuclein.

The formation of Lewy bodies may be integral to the cause of the disease rather than being an accompanying effect. Studies *in vitro* have suggested that a number of pesticides (alone or in combination with certain metals) may induce a conformational change in α-synuclein and accelerate the formation of α-synuclein fibrils (Uversky et al. 2001, 2002). Pesticides known to induce this effect are hydrophobic and include rotenone, DDT, 2,4-dichlorophenoxy-acetic acid, dieldrin, diethyldithiocarbamate, paraquat, maneb, trifluralin, parathion, and imidazoldinethione; those having no significant effect include iprodione, glyphosate, methomyl, thiuram, mevinphos, carbaryl, alachlor, thiobencarb, and also MPP^+^ (Uversky et al. 2001, 2002).

## Overall Conclusions

The epidemiologic studies suggest a relatively consistent association between exposure to pesticides and an increased risk of developing PD, despite differences in study design, case ascertainment and definition, control selection, and pesticide exposure assessment. Particular classes of pesticides found to be associated with PD include herbicides, particularly paraquat, and insecticides; evidence from case reports and case–control studies for an association with exposure to fungicides alone is equivocal. Duration of exposure has also been found to be a risk factor, with those exposed to pesticides for > 10 or 20 years being associated with an increased risk of developing PD. However, in addition to pesticides, several other risk factors are associated with an increased risk of developing PD, including rural living, well-water consumption, and farming. We found no studies that have been able to determine whether these risk factors are independent risk factors or correlated with pesticide exposure.

The toxicologic evidence suggests that, with certain routes of administration, rotenone and paraquat may have neurotoxic actions that could potentially play a role in the development of PD. These include effects on dopaminergic systems in the SN, and α-synuclein aggregation. There is also some evidence that the mechanisms of neurotoxicity associated with exposure to pyrethroids are those that would be suggestive of a role in the development of PD and that dithiocarbamates may interact with other xenobiotic agents to increase neurotoxicity. Studies on various other pesticides suggest that, while they have neurotoxic actions, they do not act on systems in the brain of relevance to PD. However, many of these studies reviewed were designed to elicit acute toxicity in order to study the mechanisms of action. We identified no study that administered pesticides at levels comparable with those encountered by pesticides users, nor were the routes of administration those that would be experienced by pesticide users (i.e., oral, inhalation, or dermal). As a result, it is difficult to interpret the relevance of such studies to humans, although the difficulty in modeling a disease such as PD is acknowledged. Of potential toxicologic importance are the few studies that reported dopaminergic neurotoxicity after combined low-level exposure to multiple environmental neurotoxicants, including paraquat and maneb, the combined effects of pesticides and metals on α-synuclein, and rotenone and lipopolysaccharide (which may be present due to inflammation or infection). For example, although PD is a disease of aging, the studies of Thiruchelvam et al. (2003) on the developmental exposure to maneb and paraquat indicate that early exposure may lead to PD-like toxic effects upon adult rechallenge. Such studies suggest that exposure to multiple low-level environmental neurotoxicants, perhaps at an early age, may be an etiologic factor in the development of PD.

Recent toxicologic studies have suggested that multiple genetic and environmental factors could be involved in the etiology of PD. Studies with transgenic mice suggest that the genetic background and expression of the α-synuclein gene may have a role to play in neurodegeneration of the SN (Thiruchelvam et al. 2004) and may also lead to increased vulnerability to the neurotoxic effects of the pesticides maneb and paraquat. There is evidence that developmental exposure to pesticides may have an increased neurodegenerative effect as well as making the SN more susceptible to subsequent adult exposure to pesticides, and that combined exposure to pesticides such as maneb and paraquat has a greater neurotoxic effect than either pesticide alone (Cory-Slechta et al. 2005). Other recent studies also suggest some interaction between the neurodegenerative effects of pesticides and inflammatory proteins produced by microglia in the SN (Gao et al. 2003, Liu and Hong 2003). These genetic and environmental factors could be considered in future epidemiologic studies of this multifactorial disease.

Most of the epidemiologic studies that we reviewed used a case–control design with relatively small numbers of cases. Pesticide exposure history was, by necessity, collected retrospectively, generally using questionnaires. Information and recall bias are inherent limitations of this type of design. The exposure assessments were also limited in their collection of information on the types of pesticides, specific chemicals, and levels of exposure experienced. Of all the studies we reviewed, the two most reliable were large case–control studies that attempted to investigate exposure to different groups of pesticides (Semchuk et al. 1992; Seidler et al. 1996).

Despite these considerations, it seems unlikely that the relatively consistent association between PD and reported exposure to pesticides observed in the epidemiology studies could be explained wholly by a combination of chance, bias and confounding, and selective reporting. The toxicologic literature indicates several areas that would benefit from further research, including the effect of exposure at different ages, early exposure and developmental changes, the role of inflammatory disease, and the potential for gene–environment interactions. Epidemiologic studies of an appropriate design and size, that collect detailed information on exposure to specific pesticides and other chemicals, including early life exposures, would be required to investigate these issues. Studies to date have not had sufficient power to disentangle the relative importance of intercorrelated risk factors and to evaluate each risk with any confidence. We are aware of several ongoing studies that are addressing some of these areas of concern.

In conclusion, the weight of evidence is sufficient to conclude that a generic association between pesticide exposure and PD exists, but it is not sufficient to conclude that this is a causal relationship or that such a relationship exists for any particular pesticide compound or combined exposure to pesticides and other exogenous toxicants. In addition, the multifactorial etiology of PD hampers unequivocally establishing the role of any individual contributory causal factor.

## Supplementary Material

Supplemental Figures and Tables
