# Supplementary material for: Pesticides and Parkinson’s Disease—Is There a Link?
Source: Environ Health Perspect. 2005 Sep 7;114(2):156–64. doi: 10.1289/ehp.8095 (PMC1367825; doi:10.1289/ehp.8095)
Supplement: Supplemental Figures and Tables [file ehp0114-000156s1.pdf]

SUPPLEMENTARY MATERIAL

Pesticides And Parkinson's Disease – Is There A Link?

Authors:

Terry P Brown,[1] Paul C Rumsby,[2] Alexander C Capleton,[1] Lesley Rushton,[1] Leonard S Levy[1]

Institution where work was performed:

MRC Institute for Environment and Health, University of Leicester, 94 Regent Road, Leicester, LE1 7DD, United Kingdom

Affiliations:

[1]MRC Institute for Environment and Health, University of Leicester, 94 Regent Road, Leicester, LE1 7DD, United Kingdom

[2]National Centre for Environmental Toxicology, WRc-NSF Ltd, Henley Road, Medmenham, Marlow, SL7 2HD, United Kingdom

Corresponding author: Alexander C Capleton

MRC Institute for Environment and Health

University of Leicester

94 Regent Road

Leicester

LE1 7DD

United Kingdom

Phone: +44 (0) 116 223 1606

Fax: +44 (0) 116 223 1601

E-mail:acc8@leicester.ac.uk

Running title: Pesticides and Parkinson's disease

Keywords: Pesticides, Parkinson's disease, literature review, toxicology, epidemiology

Abbreviations:

2,4-D	2,4-Dichlorophenoxyacetic acid
GABA	γ-Aminobutyric acid
H_2O_2	Hydrogen peroxide
NMDA	*N*-Methyl-D-aspartate
NO	Nitric oxide
PD	Parkinson's disease
ROS	Reactive oxygen species

Epidemiological evidence

Case reports and case series

Twelve cases have been reported of acute exposure to pesticides giving rise to symptoms similar to those experienced in Parkinson's disease (PD), after attempted suicide, occupational pesticide exposure in farming and house fumigation (Arima et al. 2003; Bhatt et al. 1999; Bocchetta and Corsini 1986; Lazzarino De Lorenzo 2000; Müller-Vahl et al. 1999; Sechi et al. 1992; Shahar and Andraws 2001; Stefano et al. 1989). Nine of the cases involved organophosphate pesticides, eight of which recovered with treatment (three with an anti-parkinsonism drug) and/or removal from the apparent exposure. The remaining three cases involved exposure to other pesticides over longer time periods, one of which recovered following removal from the exposure source. Two studies also describe three young cases (about 30 years of age) showing parkinsonism who had chronic exposure to the fungicide maneb, two of whom responded to treatment (Ferraz et al. 1988; Meco et al. 1994).

One case series study was identified that examined the life histories of a group of 21 young onset PD patients (Rajput et al. 1986; Rajput et al. 1987). The study found no association with young onset PD and the use of paraquat or other herbicides in agriculture, although this is consistent with current thinking that young onset PD is a primarily genetic disease (Tanner 2003).

Incidence, prevalence and mortality studies

Parkinson's disease was found to be significantly associated with the geographical distribution of pesticide usage in agriculture in one of the three prevalence studies (Barbeau et al. 1987) and in all the three mortality studies (Ritz and Yu 2000; Strickland et al. 1996; Vanacore et al. 1991). In a second prevalence study an increased risk of parkinsonism was

observed with the general use of pesticides, but not for specific pesticides (Engel et al. 2001a). In the other prevalence study and the only incidence study looking at pesticide exposure no association was found with hexachlorobenzene exposure and the chronology of major herbicide and pesticide usage, respectively (Bennett et al. 1988; Sala et al. 1999). However, other risk factors have also been associated with PD including farming as an occupation (Goldsmith et al. 1990; Goldsmith et al. 1997; Granieri et al. 1991; La Bella et al. 1990; Lee et al. 2002; Rybicki et al. 1993; Schulte et al. 1996; Wang et al. 1994a), and rural living (Ben-Shlomo et al. 1993; Bennett et al. 1988; Errea et al. 1999; Imaizumi 1995; Svenson et al. 1993; Tandberg et al. 1995), although there are some conflicting findings regarding the association with rural living (Ferraz et al. 1996; Kuopio et al. 1999b; Rybicki et al. 1993; Sethi et al. 1989; Taba and Asser 2002).

Cohort studies

Four of the five cohort studies reviewed identified farming or agriculture as an occupation (which was considered a proxy for pesticide exposure) as a significant risk factor for PD (Baldi et al. 2003; Petrovitch et al. 2002; Tüchsen and Jensen 2000; Vanacore et al. 2002). Standardised hospitalisation rates (Tüchsen et al. 2000), incidence and relative risk rates (Baldi et al. 2003; Petrovitch et al. 2002) for PD were all increased for agricultural workers. The relative risk was shown to increase with number of years of plantation work and of self-reported pesticide use (Petrovitch et al. 2002) and also with the cumulative occupational exposure to pesticides (Baldi et al. 2003). One study identified a significantly lower prevalence of PD amongst farmers compared with non-farm workers (Yesalis III et al. 1985). However, these studies should be treated with caution as other occupational groups were also identified at an increased risk of PD (Tüchsen et al. 2000), and none were designed to investigate PD risk specifically. Indeed, only one study (Vanacore et al. 2002) assembled

their cohort from a population of licensed pesticide applicators and none of the studies had an independent measure of pesticide exposure (rather, all the studies used occupation as an exposure variable). Apart from one Danish study (Tüchsen et al. 2000), the numbers of PD cases identified in each study were small (up to 116), limiting their power to detect an association. In addition the criteria used to define cases were not given, and in two studies (Baldi et al. 2003; Yesalis III et al. 1985) health status was self-reported and not clinically confirmed.

Toxicological evidence

Potential cellular mechanisms in the development of Parkinson's disease

A number of potential mechanisms involved in the death of dopaminergic neurons in the substantia nigra have been proposed, an understanding of which is important when evaluating the potential role of pesticides in PD development. Discussed in more detail below are the potential mechanisms outlined in Figure 3.

Inhibition of Complex I of the mitochondrial electron transport chain has been strongly implicated in the pathogenesis of PD. The potential involvement of Complex I inhibition was first identified when it was found that 1-methyl-4-phenylpyridine, the active metabolite of 1-methyl-4-phenyl-1,2,3,6-tetrahydropyridine, accumulates in the mitochondria of dopaminergic neurons and exerts its toxicity by inhibition of Complex I of the mitochondrial electron transport chain (Foley and Riederer 2000; Greenamyre et al. 1999; Greenamyre et al. 2001; Nicklas et al. 1992; Sherer et al. 2002). Additionally, a decrease of about 30% in Complex I activity in the substantia nigra, striatum, skeletal muscle and platelets has been noted in idiopathic PD patients without detectable structural or mitochondrial DNA changes (Foley and Riederer 2000), further suggesting a role for Complex I in PD pathogenesis.

The mechanisms by which Complex I inhibition may lead to the neurodegeneration seen in PD are yet to be fully determined. However, there is evidence that oxidative stress may have a role in PD pathogenesis, for example, brains from PD patients have been found to have elevated markers of oxidative damage (Sherer et al. 2002). The source of this oxidative stress is believed to result from the inhibition of Complex I, which leads to an increased production of reactive oxygen species (ROS). This could then result in a feed-forward cycle whereby the ROS further damage Complex I, further increasing ROS production and hence Complex I damage (Sherer et al. 2002). Reactive oxygen species (in the form of H_2O_2) are also produced

during the auto-oxidation of dopamine and during the synthesis and metabolism of dopamine by tyrosine hydroxylase and monoamine oxidase, respectively (Foley and Riederer 2000). Normally, H_2O_2 is removed by glutathione, but glutathione is present at levels lower than elsewhere in the brain and has found to be further reduced amongst PD patients. As a result, this leads to higher than normal levels of H_2O_2 in the neurons, which decompose to hydroxyl radicals via the iron mediated Fenton reaction (Bharath et al. 2002; Foley and Riederer 2000). This would require iron in the free ferrous form, which is present at high levels in the substantia nigra and has been found to be higher in PD patients compared with age matched controls (Bharath et al. 2002). Overall, this suggests an important role for oxidative stress in the development of PD.

Complex I dysfunction may also result in neurons being vulnerable to excitotoxic insults by altering adenosine triphosphate levels, by impaired calcium homeostasis or both (Sherer et al. 2002). Glutamate is the predominant excitatory neurotransmitter in the brain. It does, however, have excitotoxic properties under some conditions (Greenamyre et al. 1999). The impaired mitochondrial Complex I activity associated with PD may predispose neurons to excitotoxic cell death by removal of the Mg^{2+} blockade of the *N*-methyl-D-aspartate (NMDA) glutamate receptor. This blockade normally acts to prevent the excitotoxic stimulation of glutamate caused by an abnormal cellular influx of calcium. Without the blockade even normal cellular levels of glutamate may cause excitotoxic activation of the NMDA receptors and lead to a potentially fatal increase in intracellular calcium concentration (Sherer et al. 2002). The sequestration of calcium in the mitochondria, which may normally mitigate this effect, is decreased when electron transport is impaired (Greenamyre et al. 1999).

Nitric oxide (NO) has also been suggested as contributing to nigrostriatal injury. Inducible NO synthase is known to be increased in the substantia nigra in PD and increased NO could

also elevate local oxidative stress. Although NO is an effective free radical scavenger, it can react with the superoxide radical to form the peroxynitrite anion, a potent oxidative radical. NO also directly inhibits mitochondrial respiration (mainly at the level of Complex IV, but also at Complex I; Foley and Riederer 2000), thus adding to the oxidative stress.

Finally, three different components of Lewy bodies, wild-type human α-synuclein, parkin and ubiquitin carboxyterminal hydrolase, have been associated with genetic mutations in familial PD, which points to the possibility that altered protein conformation and/or degradation could be a key and common factor in sporadic PD. Transgenic mouse models in which α-synuclein is overexpressed show features of PD, including loss of dopaminergic nigrostriatal neurons and motor impairment (Betarbet et al. 2002a; Di Monte et al. 2002). Mice containing a doubly mutated human α-synuclein gene also showed an age-related decline in motor coordination and adverse effects on the integrity of dopamine terminals (Richfield et al. 2002).

Other pesticides

While there is evidence for neurotoxic effects of some other pesticides, all the mechanistic systems seen in PD are not consistently effected. A review of these other pesticides is presented below.

While the main neurotoxic effect of lindane is the inhibition of $GABA_A$ receptors (Rivera et al. 1998), other neurotransmitter levels were increased including dopaminergic neurons in the substantia nigra (Artigas et al. 1988). It is not clear whether the observed changes in other neurotransmitter pathways are due to the release of the inhibitory action of GABA.

When administered to mice the organophosphate **c**hlorpyrifos resulted in a small decrease in striatal dopamine uptake, a decrease in mitochondrial function, an increase in dopamine

turnover and a decrease in open field behaviour (Karen et al. 2001). Although the organophosphate dichlorvos is a directly acting inhibitor of acetylcholinesterase, marked changes to the dopaminergic neurotransmitter system, including decreased dopamine binding and increased activity of tyrosine hydroxylase and dopamine-β-hydroxylase, are also seen (Choudhary et al. 2002). The authors suggested that alterations in the dopamine system may be a causative mechanism behind the behavioural and functional changes associated with delayed organophosphate neurotoxicity.

At high doses carbaryl has been shown to induce tremor, which can be reduced by prior treatment with L-dopa (Rigon et al. 1994). Carbaryl potentiated the catalepsy induced by the striatal dopaminergic receptor blocker, haloperidol. This led the authors to suggest that the effects of carbaryl involved a disturbance of the balance between cholinergic and dopaminergic systems.

Injection of 2,4-dichlorophenoxyacetic acid (2,4-D) into one striatum of a rat produced a marked depression in locomotor activity and circling behaviour and an increase in dopamine metabolism (Bortolozzi et al. 2001) indicated neurotoxicity in the basal ganglia. In pregnant rats treated with a 1:1 mixture of 2,4-D and 2,4,5-trichlorophenoxyacetic acid there was delayed ontogeny of brain dopamine (but not noradrenaline), together with a delay in the development of certain neurobehaviour in pups (Mohammad and St Omer 1985).

A heightened locomotor and stereotype response was observed in rats receiving the antifungal agent, triadimefon, at very high doses (Hill et al. 2000), primarily through the potentiation of dopamine activity. There was an increase in dopamine uptake and release in the striatum and nucleus accumbens.

References

Adler CH. 1999. Differential diagnosis of Parkinson's disease. Med Clin North Am 83:349-367.

Alam M, Schmidt WJ. 2002. Rotenone destroys dopaminergic neurons and induces parkinsonian symptoms in rats. Behav Brain Res 136:317-324.

Ansari RA, Husain K, Gupta PK. 1987. Endosulfan toxicity influence on biogenic amines of rat brain. J Environ Biol 8:229-236.

Arima H, Sobue K, So M, Morishima T, Ando H, Katsuya H. 2003. Transient and reversible parkinsonism after acute organophosphate poisoning. J Toxicol Clin Toxicol 41:60-70.

Artigas F, Martínez E, Camón L, Farré ER. 1988. Synthesis and utilization of neurotransmitters: Actions of subconvulsant doses of hexachlorocyclohexane isomers on brain monoamines. Toxicology 49:49-55.

Bagetta G, Corasaniti MT, Iannone M, Nisticò G, Stephenson JD. 1992. Production of limbic motor seizures and brain damage by systemic and intracerebral injections of paraquat in rats. Pharmacol Toxicol 71:443-448.

Baldi I, Lebailly P, Mohammed-Brahim B, Letenneur L, Dartigues J-F, Brochard P. 2003. Neurodegenerative diseases and exposure to pesticides in the elderly. Am J Epidemiol 157:409-414.

Barbeau A, Roy M, Bernier G, Campanella G, Paris S. 1987. Ecogenetics of Parkinson's disease: Prevalence and environmental aspects in rural areas. Can J Neurol Sci 14:36-41.

Barlow BK, Thiruchelvam MJ, Bennice L, Cory-Slechta DA, Ballatori N, Richfield EK. 2003. Increased synaptosomal dopamine content and brain concentration of paraquat produced by selective dithiocarbamates. J Neurochem 85:1075-1086.

Behari M, Srivastava AK, Das RR, Pandey RM. 2001. Risk factors of Parkinson's disease in Indian patients. J Neurol Sci 190:49-55.

Ben-Shlomo Y, Finnan F, Allwright S, Davey SG. 1993. The epidemiology of Parkinson's disease in the Republic of Ireland: Observations from routine data sources. Ir Med J 86:190-191.

Bennett V, Rajput AH, Uitti RJ. 1988. An epidemiological survey of agricultural chemicals and incidence of Parkinson's disease. Neurology 38:349.

Betarbet R, Sherer TB, Di Monte DA, Greenamyre JT. 2002b. Mechanistic approaches to Parkinson’s disease pathogenesis. Brain Pathol 12:499–510.

Betarbet R, Sherer TB, Greenamyre JT. 2002a. Animal models of Parkinson's disease. Bioessays 24:308-318.

Betarbet R, Sherer TB, MacKenzie G, Garcia-Osuna M, Panov AV, Greenamyre JT. 2000. Chronic systemic pesticide exposure reproduces features of Parkinson's disease. Nat Neurosci 3:1301-1306.

Bharath S, Hsu M, Kaur D, Rajagopalan S, Andersen JK. 2002. Glutathione, iron and Parkinson's disease. Biochem Pharmacol 64:1037-1048.

Bhatt MH, Elias MA, Mankodi AK. 1999. Acute and reversible parkinsonism due to organophosphate pesticide intoxication: Five cases. Neurology 52:1467-1471.

Bloomquist JR, Kirby ML, Castagnoli K, Miller GW. 1999. Effects of heptachlor exposure on neurochemical biomarkers of parkinsonism. In: Progress in Neuropharmacology and Neurotoxicology of Pesticides and Drugs. (Beadle DJ, ed). Cambridge, UK:Royal Society of Chemistry, 203.

Bocchetta A, Corsini GU. 1986. Parkinson's disease and pesticides. Lancet 2:1163.

Bortolozzi A, Evangelista de Duffard AM, Dajas F, Duffard R, Silveira R. 2001. Intracerebral administration of 2,4-dichlorophenoxyacetic acid induces behavioral and neurochemical alterations in the rat brain. Neurotoxicology 22:221-232.

Butterfield PG, Valanis BG, Spencer PS, Lindeman CA, Nutt JG. 1993. Environmental antecedents of young-onset Parkinson's disease. Neurology 43:1150-1158.

Calò M, Iannone M, Passafaro M, Nisticò G. 1990. Selective vulnerability of hippocampal CA3 neurones after microinfusion of paraquat into the rat substantia nigra or into the ventral tegmental area. J Comp Pathol 103:73-78.

Chan DKY, Woo J, Ho SC, Pang CP, Law LK, Ng PW, Hung WT, Kwok T, Hui E, Orr K, Leung MF, Kay R. 1998. Genetic and environmental risk factors for Parkinson's disease in a Chinese population. J Neurol Neurosurg Psych 65:781-784.

Chaturvedi S, Østbye T, Stoessl AJ, Merskey H, Hachinski V. 1995. Environmental exposures in elderly Canadians with Parkinson's disease. Can J Neurol Sci 22:232-234.

Choudhary S, Raheja G, Gupta V, Gill KD. 2002. Possible involvement of dopaminergic neurotransmitter system in dichlorvos induced delayed neurotoxicity. J Biochem Mol Biol Biophys 6:29-36.

Corasaniti MT, Bagetta G, Rodinò P, Gratteri S, Nisticò G. 1992. Neurotoxic effects induced by intracerebral and systemic injection of paraquat in rats. Hum Exp Toxicol 11:535-539.

Corasaniti MT, Strongoli MC, Rotiroti D, Bagetta G, Nisticò G. 1998. Paraquat: A useful tool for the *in vivo* study of mechanisms of neuronal cell death. Pharmacol Toxicol 83:1-7.

Corrigan FM, Murray L, Wyatt CL, Shore RF. 1998. Diorthosubstituted polychlorinated biphenyls in caudate nucleus in Parkinson's disease. Exp Neurol 150:339-342.

Corrigan FM, Wienburg CL, Shore RF, Daniel SE, Mann D. 2000. Organochlorine insecticides in substantia nigra in Parkinson's disease. J Toxicol Environ Health A 59:229-234.

Cory-Slechta DA, Thiruchelvam M, Barlow BK, Richfield EK (2005) Developmental pesticide models of the Parkinson's disease phenotype. Environ Health Perspect doi: 10.1289/ehp.7570

De Gori N, Froio F, Strongoli MC, De Francesco A, Calò M, Nisticò G. 1988. Behavioural and electrocortical changes induced by paraquat after injection in specific areas of the brain of the rat. Neuropharmacology 27:201-208.

de Rijk MC, Launer LJ, Berger K, Breteler MMB, Dartigues J-F, Baldereschi M, Fratiglioni L, Lobo A, Martinez-Lage J, Trenkwalder C, Hofman A. 2000. Prevalence of Parkinson's disease in Europe: A collaborative study of population-based cohorts. Neurology 54 (Suppl 5):S21-S23.

Di Monte DA, Lavasani M, Manning-Bog AB. 2002. Environmental factors in Parkinson's disease. Neurotoxicology 23:487-502.

Duzcan F, Zencir M, Ozdemir F, Cetin GO, Bagci H, Heutink P, Bonifati V, Sahiner T. 2003. Familial influence on Parkinsonism in a rural area of Turkey (Kizilcaboluk-Denizli): A community-based case-control study. Mov Disord 18:799-804.

Engel LS, Checkoway H, Keifer MC, Seixas NS, Longstreth WTJ, Scott KC, Hudnell K, Anger WK, Camicioli R. 2001a. Parkinsonism and occupational exposure to pesticides. Occup Environ Med 58:582-589.

Engel LS, Seixas NS, Keifer MC, Longstreth WTJ, Checkoway H. 2001b. Validity study of self-reported pesticide exposure among orchardists. J Expo Anal Environ Epidemiol 11:359-368.

Errea JM, Ara JR, Aibar C, de Pedro-Cuesta J. 1999. Prevalence of Parkinson's disease in lower Aragon, Spain. Mov Disord 14:596-604.

Fahn S, Elton R. 1987. Unified Parkinson's disease rating scale. In: Recent Developments in Parkinson's Disease, Volume 2. (Fahn S, Marsden CD, Calne DB, Goldstein M, eds). USA:Macmillan Health Care Information.

Fall P-A, Fredrikson M, Axelson O, Granérus A-K. 1999. Nutritional and occupational factors influencing the risk of Parkinson's disease: A case-control study in Southeastern Sweden. Mov Disord 14:28-37.

Falope ZF, Osuntokun BO, Ogunniyi A. 1992. Risk factors for Parkinson's disease in Nigerian Africans: A case-control study. J Trop Geograph Neurol 2:177-180.

Ferraz HB, Andrade LAF, Tumas V, Calia LC, Borges V. 1996. Rural or urban living and Parkinson's disease. Arq Neuropsiquiatr 54:37-41.

Ferraz HB, Bertolucci PHF, Pereira JS, Lima JGC, Andrade LAF. 1988. Chronic exposure to the fungicide maneb may produce symptoms and signs of CNS manganese intoxication. Neurology 38:550-553.

Firestone JA, Smith-Weller T, Franklin G, Swanson P, Longstreth WT, Checkoway H. 2005. Pesticides and risk of Parkinson’s disease. A population-based case-control study. Arch Neurol 62(1):91-95.

Fleming L, Mann JB, Bean J, Briggle T, Sanchez-Ramos JR. 1994. Parkinson's disease and brain levels of organochlorine pesticides. Ann Neurol 36:100-103.

Foley P, Riederer P. 2000. Influence of neurotoxins and oxidative stress on the onset end progression of Parkinson's disease. J Neurol 247(Suppl 2):82-94.

Foltynie T, Sawcer S, Brayne C, Barker RA. 2002. The genetic basis of Parkinson's disease. J Neurol Neurosurg Psych 73:363-370.

Fredriksson A, Fredriksson M, Eriksson P. 1993. Neonatal exposure to paraquat or MPTP induces permanent changes in striatum dopamine and behavior in adult mice. Toxicol Appl Pharmacol 122:258-264.

Gao H-M, Hong J-S, Zhang W, Liu B. 2002. Distinct role for microglia in rotenone-induced degeneration of dopaminergic neurons. J Neurosci 22: 782-790.

Gao H-M, Hong J-S, Zhang W, Liu B. 2003. Synergistic dopaminergic neurotoxicity of the pesticide rotenone and inflammogen lipopolysaccharide: Relevance to the etiology of Parkinson's disease. J Neurosci 23:1228-1236.

Golbe LI, Farrell TM, Davis PH. 1990. Follow-up study of early-life protective and risk factors in Parkinson's disease. Mov Disord 5:66-70.

Goldman SM, Tanner C. 1998. Etiology of Parkinson's disease. In: Parkinson's Disease and Movement Disorders. (Jankovic J, Tolosa E, eds). London, UK:Williams and Wilkins.

Goldsmith JR, Herishanu Y, Abarbanel JM, Weinbaum Z. 1990. Clustering of Parkinson's disease points to environmental etiology. Arch Environ Health 45:88-94.

Goldsmith JR, Herishanu YO, Podgaietski M, Kordysh E. 1997. Dynamics of parkinsonism-Parkinson's disease in residents of adjacent kibbutzim in Israel's Negev. Environ Res 73:156-161.

Gorell JM, Johnson CC, Rybicki BA, Peterson EL, Richardson RJ. 1998. The risk of Parkinson's disease with exposure to pesticides, farming, well water, and rural living. Neurology 50:1346-1350.

Granieri E, Carreras M, Casetta I, Govoni V, Tola MR, Paolino E, Monetti VC, De Bastiani P. 1991. Parkinson's disease in Ferrara, Italy, 1967 through 1987. Arch Neurol 48:854-857.

Greenamyre JT, MacKenzie G, Peng T-I, Stephans SE. 1999. Mitochondrial dysfunction in Parkinson's disease. Biochem Soc Symp 66:85-97.

Greenamyre JT, Sherer TB, Betarbet R, Panov AV. 2001. Complex I and Parkinson's disease. IUBMB Life 52:135-141.

Herishanu YO, Medvedovski M, Goldsmith JR, Kordysh E. 2001. A case-control study of Parkinson's disease in urban population of southern Israel. Can J Neurol Sci 28:144-147.

Hernán MA, Takkouche B, Caamaño-Isorna F, Gestal-Otero JJ. 2002. A meta-analysis of coffee drinking, cigarette smoking, and the risk of Parkinson’s disease. Ann Neurol 52:276-284.

Hertzman C, Wiens M, Bowering D, Snow B, Calne D. 1990. Parkinson's disease: A case-control study of occupational and environmental risk factors. Am J Ind Med 17:349-355.

Hertzman C, Wiens M, Snow B, Kelly S, Calne D. 1994. A case-control study of Parkinson's disease in a horticultural region of British Columbia. Mov Disord 9:69-75.

Hill D, Ikaiddi M, Mazzio E, Soliman KFA. 2000. The neurochemical basis for the behavioral effects of triadimefon. Ann N Y Acad Sci 914:336-353.

Ho SC, Woo J, Lee CM. 1989. Epidemiologic study of Parkinson's disease in Hong Kong. Neurology 39:1314-1318.

Hoehn MM, Yahr M. 1998. Parkinsonism: Onset, progression, and mortality. Neurology 50:318-341.

Hoppin JA, Yucel F, Dosemeci M, Sandler DP. 2002. Accuracy of self-reported pesticide use duration information from licensed pesticide applicators in the Agricultural Health Study. J Expo Anal Environ Epidemiol 12:313-318.

Hubble JP, Cao T, Hassanein RES, Neuberger JS, Koller WC. 1993. Risk factors for Parkinson's disease. Neurology 43:-1697.

Hughes AJ, Ben-Shlomo Y, Daniel SE, Lees AJ. 1992a. What features improve the accuracy of the clinical diagnosis in Parkinson's disease: A clinicopathologic study. Neurology 42:1142-1146.

Hughes AJ, Daniel SE, Kilford L, Lees AJ. 1992b. Accuracy of clinical diagnosis of idiopathic Parkinson's disease: A clinico-pathological study of 100 cases. J Neurol Neurosurg Psych 55:181-184.

Husain R, Gupta A, Khanna VK, Seth PK. 1991. Neurotoxicological effects of a pyrethroid formulation, fenvalerate in rats. Res Commun Chem Pathol Pharmacol 73:111-114.

Iannone M, Calò M, Rispoli V, Sancesario G, Nisticò G. 1988. Neuropathological lesions after microinfusion of paraquat and MPP^+ into different areas of the rat brain. Acta Neurol 43:313-321.

Iannone M, Ciriolo MR, Rotilio G, Nistico G. 1991. Intra-nigral infusion of Cu-free superoxide dismutase prevents paraquat-induced behavioral stimulation and ECoG epileptogenic discharges in rats. Neuropharmacology 30:893-898.

Imaizumi Y. 1995. Geographical variations in mortality from Parkinson's disease in Japan, 1977-1985. Acta Neurol Scand 91:311-316.

Jiménez-Jiménez FJ, Mateo D, Giménez-Roldán S. 1992. Exposure to well water and pesticides in Parkinson's disease: A case-control study in the Madrid area. Mov Disord 7:149-152.

Kamel F, Tanner CM, Hoppin JA, Umbach DM, Goldman SM, Aston DA, Ross GW, Alavanja MCR, Blair A, Langston JW, Sandler DP. 2001. Pilot study of Parkinson's disease (PD) in the agricultural health study (AHS). Neurotoxicology 22:883-884.

Karen DJ, Li W, Harp PR, Gillette JS, Bloomquist JR. 2001. Striatal dopaminergic pathways as a target for the insecticides permethrin and chlorpyrifos. Neurotoxicology 22:811-817.

Kirby ML, Barlow RL, Bloomquist JR. 2001. Neurotoxicity of the organochlorine insecticide heptachlor to murine striatal dopaminergic pathways. Toxicol Sci 61:100-106.

Kirby ML, Castagnoli K, Bloomquist JR. 1999. *In vivo* effects of deltamethrin on dopamine neurochemistry and the role of augmented neurotransmitter release. Pesticide Biochem Physiol 65:160-168.

Kirkey KL, Johnson CC, Rybicki BA, Peterson EL, Kortsha GX, Gorell JM. 2001. Occupational categories at risk for Parkinson's disease. Am J Ind Med 39:564-571.

Kitazawa M, Anantharam V, Kanthasamy AG. 2001. Dieldrin-induced oxidative stress and neurochemical changes contribute to apoptopic cell death in dopaminergic cells. Free Radic Biol Med 31:1473-1485.

Koller W, Vetere-Overfield B, Gray C, Alexander C, Chin T, Dolezal J, Hassanein R, Tanner C. 1990. Environmental risk factors in Parkinson's disease. Neurology 40:1218-1221.

Kuopio A-M, Marttila RJ, Helenius H, Rinne UK. 1999a. Environmental risk factors in Parkinson's disease. Mov Disord 14:928-939.

Kuopio AM, Marttila RJ, Helenius H, Rinne UK. 1999b. Changing epidemiology of Parkinson's disease in southwestern Finland. Neurology 52:302-308.

La Bella V, Brighina F, Piccoli F. 1990. An epidemiological study on Parkinson's disease in a rural area: The province of Trapani, Italy. J Neurol 237 (Suppl 1):S65.

Langston JW, Ballard PA, Tetrud JW, Irwin I. 1983. Chronic parkinsonism in humans due to a product of meperidine-analog synthesis. Science 219:979-980.

Lazzarino De Lorenzo LG. 2000. Chemical environmental pollution and nervous system. Occupational and environmental risk factors for Parkinson's disease and parkinsonisms. Int J Med Biol Environ 28:149-154.

Lee E, Burnett CA, Lalich N, Cameron LL, Sestito JP. 2002. Proportionate mortality of crop and livestock farmers in the United States, 1984-1993. Am J Ind Med 42:410-420.

Liou HH, Tsai MC, Chen CJ, Jeng JS, Chang YC, Chen SY, Chen R. 1997. Environmental risk factors and Parkinson's disease: A case-control study in Taiwan. Neurology 48:1583-1588.

Litvan I, Agid Y, Jankovic J, Goetz C, Brandel JP, Lai EC, Wenning G, D'Olhaberriague L, Verny M, Chaudhuri KR, McKee A, Jellinger K, Bartko JJ, Mangone CA, Pearce RKB. 1996. Accuracy of clinical criteria for the diagnosis of progressive supranuclear palsy (Steele-Richardson-Olszewski syndrome). Neurology 46:922-930.

Litvan I, Bhatia KP, Burn DJ, Goetz CG, Lang AE, McKeith I, Quinn N, Sethi KD, Shults C, Wenning GK. 2003. SIC Task Force appraisal of clinical diagnostic criteria for Parkinsonian disorders. Mov Disord 18:467-486.

Litvan I, MacIntyre A, Goetz CG, Wenning GK, Jellinger K, Verny M, Bartko JJ, Jankovic J, McKee A, Brandel JP, Chaudhuri KR, Lai EC, D'Olhaberriague L, Pearce RK, Agid Y. 1998. Accuracy of the clinical diagnoses of Lewy body disease, Parkinson disease, and dementia with Lewy bodies: A clinicopathologic study. Arch Neurol 55:969-978.

Liu B, Hong J-S. 2003. Role of microglia in inflammation-mediated neurodegenerative diseases: mechanisms and strategies for therapeutic intervention. J Pharm Exp Ther 304:1-7.

Malaviya M, Husain R, Seth PK, Husain R. 1993. Perinatal effects of two pyrethroid insecticides on brain neurotransmitter function in the neonatal rat. Vet Hum Toxicol 35:119-122.

Manning-Bog AB, McCormack AL, Li J, Uversky VN, Fink AL, Di Monte DA. 2002. The herbicide paraquat causes up-regulation and aggregation of α−synuclein in mice. J Biol Chem 277:1641-1644.

McCann SJ, LeCouteur DG, Green AC, Brayne C, Johnson AG, Chan D, McManus ME, Pond SM. 1998. The epidemiology of Parkinson's disease in an Australian population. Neuroepidemiology 17:310-317.

McCormack AL, Thiruchelvam M, Manning-Bog AB, Thiffault C, Di Monte DA, Cory-Slechta DA, Langston JW. 2002. Environmental risk factors and Parkinson's disease: Selective degeneration of nigral dopaminergic neurons caused by the herbicide paraquat. Neurobiol Dis 10:119-127.

McGrew DM, Irwin I, Langston JW. 2000. Ethylenebisdithiocarbamate enhances MPTP-induced striatal dopamine depletion in mice. Neurotoxicology 21:309-312.

Meco G, Bonifati V, Vanacore N, Fabrizo E. 1994. Parkinsonsim after chronic exposure to the fungicide maneb (manganese ethylene-bis-dithocarbamate). Scand J Work Environ Health 20(4):301-305.

Menegon A, Board PG, Blackburn AC, Mellick GD, Le Couteur DG. 1998. Parkinson's disease, pesticides, and glutathione transferase polymorphisms. Lancet 352:1344-1346.

Miller DB, Reinhard JF, Jr., Daniels AJ, O'Callaghan JP. 1991. Diethyldithiocarbamate potentiates the neurotoxicity of in vivo 1-methyl-4-phenyl-1,2,3,6-tetrahydropyridine and of in vitro 1-methyl-4-phenylpyridinium. J Neurochem 57:541-549.

Miller GW, Kirby ML, Levey AI, Bloomquist JR. 1999. Heptachlor alters expression and function of dopamine transporters. Neurotoxicology 20:631-637.

Mohammad FK, St Omer VEV. 1985. Developing rat brain monoamine levels following in utero exposure to a mixture of 2 4-dichlorophenoxyacetic and 2,4,5-trichlorophenoxyacetic acids. Toxicol Letts 29:215-223.

Morano A, Jiménez-Jiménez FJ, Molina JA, Antolin MA. 1994. Risk-factors for Parkinson's disease: Case-control study in the province of Cáceres, Spain. Acta Neurol Scand 89:164-170.

Morato GS, Lemos T, Takahashi RN. 1989. Acute exposure to maneb alters some behavioral functions in the mouse. Neurotoxicol Teratol 11:421-425.

Morens DM, Davis JW, Grandinetti A, Ross GW, Popper JS, White LR. 1996. Epidemiologic observations on Parkinson's disease: Incidence and mortality in a prospective study of middle-aged men. Neurology 46:1044-1050.

Müller-Vahl KR, Kolbe H, Dengler R. 1999. Transient severe parkinsonism after acute organophosphate poisoning. J Neurol Neurosurg Psych 66:253-254.

Naylor JL, Widdowson PS, Simpson MG, Farnworth M, Ellis MK, Lock EA. 1995. Further evidence that the blood/brain barrier impedes paraquat entry into the brain. Hum Exp Toxicol 14:587-594.

Nelson LM, Van den Eeden SK, Tanner CM, Efird JT, Bernstein AL. 2000. Home pesticide exposure and the risk of Parkinson's disease. Neurology 54:A472-A473.

Nicklas WJ, Saporito M, Basma A, Geller HM, Heikkila RE. 1992. Mitochondrial mechanisms of neurotoxicity. Ann N Y Acad Sci 648:28-36.

Niehaus I, Lange JH. 2003. Endotoxin: Is it an environmental factor in the cause of Parkinson's disease? Occup Environ Med 60:378-382.

Paul V, Balasubramaniam E. 1997. Effects of single and repeated administration of endosulfan on behaviour and its interaction with centrally acting drugs in experimental animals: A mini review. Environ Toxicol Pharmacol 3:151-157.

Petrovitch H, Ross GW, Abbott RD, Sanderson WT, Sharp DS, Tanner CM, Masaki KH, Blanchette PL, Popper JS, Foley D, Launer L, White LR. 2002. Plantation work and risk of Parkinson disease in a population-based longitudinal study. Arch Neurol 59:1787-1792.

Preux PM, Condet A, Anglade C, Druet-Cabanac M, Debrock C, Macharia W, Couratier P, Boutros-Toni F, Dumas M. 2000. Parkinson's disease and environmental factors. Matched case-control study in the Limousin region, France. Neuroepidemiology 19:333-337.

Priyadarshi A, Khuder SA, Schaub EA, Priyadarshi SS. 2001. Environmental risk factors and Parkinson's disease: a metaanalysis. Environ Res 86:122-127.

Priyadarshi A, Khuder SA, Schaub EA, Shrivastava S. 2000. A meta-analysis of Parkinson's disease and exposure to pesticides. Neurotoxicology 21:435-440.

Purkerson-Parker S, McDaniel KL, Moser VC. 2001. Dopamine transporter binding in the rat striatum is increased by gestational, perinatal, and adolescent exposure to heptachlor. Toxicol Sci 64:216-223.

Rajput AH, Uitti RJ, O'Donnell K, Stern W, Laverty W, O'Donnell D, Yuen WK, Dua A. 1987. Geography, drinking water chemistry, pesticides and herbicides and the etiology of Parkinson's disease. Can J Neurol Sci 14 (Suppl 3):414-418.

Rajput AH, Uitti RJ, Stern W, Laverty W. 1986. Early onset Parkinson's disease in Saskatchewan: Environmental considerations for etiology. Can J Neurol Sci 13:312-316.

Richfield EK, Thiruchelvam MJ, Cory-Slechta DA, Wuertzer C, Gainetdinov RR, Caron MG, Di Monte DA, Federoff HJ. 2002. Behavioural and neurochemical effects of wild-type and mutated human α-synuclein in transgenic mice. Exp Neurol 175:35-48.

Rigon AR, Reis M, Takahashi RN. 1994. Effects of carbaryl on some dopaminergic behaviors in rats. Gen Pharmacol 25:1263-1267.

Ritz B, Yu F. 2000. Parkinson's disease mortality and pesticide exposure in California 1984-1994. Int J Epidemiol 29:323-329.

Rivera S, Rosa R, Martínez E, Sunol C, Serrano MT, Vendrell M, Rodríguez-Farre E, Sanfeliu C. 1998. Behavioral and monoaminergic changes after lindane exposure in developing rats. Neurotoxicol Teratol 20:155-160.

Rybicki BA, Johnson CC, Uman J, Gorell JM. 1993. Parkinson's disease mortality and the industrial use of heavy metals in Michigan. Mov Disord 8:87-92.

Sala M, Sunyer J, Otero R, Santiago-Silva M, Ozalla D, Herrero C, To-Figueras J, Kogevinas M, Anto JM, Camps C, Grimalt J. 1999. Health effects of chronic high exposure to hexachlorobenzene in a general population sample. Arch Environ Health 54:102-109.

Sanchez-Ramos J, Facca A, Basit A, Song S. 1998. Toxicity of dieldrin for dopaminergic neurons in mesencephalic cultures. Exp Neurol 150:263-271.

Sanchez-Ramos JR, Hefti F, Weiner WJ. 1987. Paraquat and Parkinson's disease. Neurology 37:728.

Schapira AHV. 1999. Parkinson's disease. Br Med J 318:311-314.

Schoenberg BS, Osuntokun BO, Adeuja AO, Bademosi O, Nottidge V, Anderson DW, Haerer AF. 1988. Comparison of the prevalence of Parkinson's disease in black populations in the rural United States and in rural Nigeria: Door-to-door community studies. Neurology 38:645-646.

Schulte PA, Burnett CA, Boeniger MF, Johnson J. 1996. Neurodegenerative diseases: Occupational occurrence and potential risk factors, 1982 through 1991. Am J Public Health 86:1281-1288.

Sechi GP, Agnetti V, Piredda M, Canu M, Deserra F, Omar HA, Rosati G. 1992. Acute and persistent parkinsonism after use of diquat. Neurology 42:261-263.

Seidler A, Hellenbrand W, Robra B-P, Vieregge P, Nischan P, Joerg J, Oertel WH, Ulm G, Schneider E. 1996. Possible environmental, occupational, and other etiologic factors for Parkinson's disease: A case-control study in Germany. Neurology 46:1275-1284.

Semchuk KM, Love EJ. 1995. Effects of agricultural work and other proxy-derived case-control data on Parkinson's disease risk estimates. Am J Epidemiol 141:747-754.

Semchuk KM, Love EJ, Lee RG. 1992. Parkinson's disease and exposure to agricultural work and pesticide chemicals. Neurology 42:1328-1335.

Semchuk KM, Love EJ, Lee RG. 1993. Parkinson's disease: A test of the multifactorial etiologic hypothesis. Neurology 43:1173-1180.

Seth PK, Saidi NF, Agrawal AK, Anand M. 1986. Neurotoxicity of endosulfan in young and adult rats. Neurotoxicology 7:623-635.

Sethi KD, Meador KJ, Loring D, Meador MP. 1989. Neuroepidemiology of Parkinson's disease: Analysis of mortality data for the U.S.A. and Georgia. Int J Neurosci 46:87-92.

Shahar E, Andraws J. 2001. Extra-pyramidal parkinsonism complicating organophosphate insecticide poisoning. Europ J Paediatr Neurol 5:261-264.

Sherer TB, Betarbet R, Greenamyre JT. 2002. Environment, mitochondria, and Parkinson's disease. Neuroscientist 8:192-197.

Shimizu K, Matsubara K, Ohtaki K, Fujimaru S, Saito O, Shiono H. 2003. Paraquat induces long-lasting dopamine overflow through the excitotoxic pathway in the striatum of freely moving rats. Brain Res 976:243-252.

Shimizu K, Ohtaki K, Matsubara K, Aoyama K, Uezono T, Saito O, Suno- M, Ogawa K, Hayase N, Kimura K, Shiono H. 2001. Carrier-mediated processes in blood--brain barrier penetration and neural uptake of paraquat. Brain Res 906:135-142.

Smargiassi A, Mutti A, De Rosa A, De Palma G, Negrotti A, Calzetti S. 1998. A case-control study of occupational and environmental risk factors for Parkinson's disease in the Emilia-Romagna region of Italy. Neurotoxicology 19:709-712.

Soleo L, Defazio G, Scarselli R, Zefferino R, Livrea P, Foa V. 1996. Toxicity of fungicides containing ethylene-bis-dithiocarbamate in serumless dissociated mesencephalic-striatal primary coculture. Arch Toxicol 70:678-682.

Stedeford T, Cardozo-Pelaez F, Nemeth N, Song S, Harbison RD, Sanchez-Ramos J. 2001. Comparison of base-excision repair capacity in proliferating and differentiated PC 12 cells following acute challenge with dieldrin. Free Radic Biol Med 31:1272-1278.

Stefano E, Casali C, Caporali M, Sancesario G, Morocutti C. 1989. Parkinson disease in farm workers. Ital J Neurol Sci 10:379.

Stern M, Dulaney E, Gruber SB, Golbe L, Bergen M, Hurtig H, Gollomp S, Stolley P. 1991. The epidemiology of Parkinson's disease. A case-control study of young-onset and old-onset patients. Arch Neurol 48:903-907.

Strickland D, Bertoni JM, Pfeiffer RF. 1996. Descriptive epidemiology of Parkinson's disease through proxy measures. Can J Neurol Sci 23:279-284.

Svenson LW, Platt GH, Woodhead SE. 1993. Geographic variations in the prevalence rates of Parkinson's disease in Alberta. Can J Neurol Sci 20:307-311.

Taba P, Asser T. 2002. Prevalence of Parkinson's disease in Estonia. Acta Neurol Scand 106:276-281.

Takahashi RN, Rogerio R, Zanin M. 1989. Maneb enhances MPTP neurotoxicity in mice. Res Commun Chem Pathol Pharmacol 66:167-170.

Tandberg E, Larsen JP, Nessler EG, Riise T, Aarli JA. 1995. The epidemiology of Parkinson's disease in the county of Rogaland, Norway. Mov Disord 10:541-549.

Tanner CM. 2003. Is the cause of Parkinson's disease environmental or hereditary? Evidence from twin studies. In: Parkinson's Disease: Advances in Neurology, Volume 91. (Gordin A, Kaakkola S, Teräväinen H, eds). Philadelphia, USA:Lippincott Williams and Wilkins, 133-142.

Tanner CM, Chen B, Wang W, Peng M, Liu Z, Liang X, Kao LC, Gilley DW, Goetz CG, Schoenberg BS. 1989. Environmental factors and Parkinson's disease: A case-control study in China. Neurology 39:660-664.

Tawara T, Fukushima T, Hojo N, Isobe A, Shiwaku K, Setogawa T, Yamane Y. 1996. Effects of paraquat on mitochondrial electron transport system and catecholamine contents in rat brain. Arch Toxicol 70:585-589.

Taylor CA, Saint-Hilaire MH, Cupples LA, Thomas CA, Burchard AE, Feldman RG, Myers RH. 1999. Environmental, medical, and family history risk factors for Parkinson's disease: A New England-based case control study. Am J Med Genet 88:742-749.

Teitelbaum SL. 2002. Questionnaire assessment of nonoccupational pesticide exposure in epidemiologic studies of cancer. J Expo Anal Environ Epidemiol 12:373-380.

Thiruchelvam M, Brockel BJ, Richfield EK, Baggs RB, Cory-Slechta DA. 2000a. Potentiated and preferential effects of combined paraquat and maneb on nigrostriatal dopamine systems: Environmental risk factors for Parkinson's disease? Brain Res 873:225-234.

Thiruchelvam M, McCormack A, Richfield EK, Baggs RB, Tank AW, Di Monte DA, Cory-Slechta DA. 2003. Age-related irreversible progressive nigrostriatal dopaminergic neurotoxicity in the paraquat and maneb model of the Parkinson's disease phenotype. European Journal of Neuroscience 18:589-600.

Thiruchelvam MJ, Powers JM, Cory-Slechta DA, Richfield EK. 2004. Risk factors for dopaminergic neuron loss in human α-synuclein transgenic mice. Eur J Neurosci 19:845-854.

Thiruchelvam M, Richfield EK, Baggs RB, Tank AW, Cory-Slechta DA. 2000b. The nigrostriatal dopaminergic system as a preferential target of repeated exposures to combined paraquat and maneb: Implications for Parkinson's disease. J Neurosci 20:9207-9214.

Thiruchelvam M, Richfield EK, Goodman BM, Cory-Slechta DA, Baggs RB. 2002. Developmental exposure to the pesticides paraquat and maneb and the Parkinson's disease phenotype. Neurotoxicology 22:872-873.

Tüchsen F, Jensen AA. 2000. Agricultural work and the risk of Parkinson's disease in Denmark, 1981-1993. Scand J Work Environ Health 26:359-362.

Twelves D, Perkins KSM, Counsell C. 2003. Systematic review of incidence studies of Parkinson's disease. Mov Disord 18:19-31.

Uversky VN, Li J, Bower K, Fink AL. 2002. Synergistic effects of pesticides and metals on the fibrillation of α-synuclein: Implications for Parkinson's disease. Neurotoxicology 23:527-536.

Uversky VN, Li J, Fink AL. 2001. Pesticides directly accelerate the rate of α-synuclein fibril formation: A possible factor in Parkinson's disease. FEBS Lett 500:105-108.

Vanacore N, Bonifati V, Fabrizio E, Modarelli FT, Meco G. 1991. Epidemiologic study of geographical correlation between consumption of herbicides and paraquat and Parkinson's disease (PD) mortality rates in Italy (period 1969 to 1987). Neurology 41 (suppl 1):307.

Vanacore N, Nappo A, Gentile M, Brustolin A, Palange S, Liberati A, Di Rezze S, Caldora G, Gasparini M, Benedetti F, Bonifati V, Forastiere F, Quercia A, Meco G. 2002. Evaluation of risk of Parkinson's disease in a cohort of licensed pesticide users. Neurol Sci 23 (suppl 2):S119-S120.

Vidal J-S, Elbaz A, Clavel J, Delemotte B, Alpérovitch A, Tzouri C. 2002. Exposure to pesticides and Parkinson's disease: A community-based case-control study among a population characterised by a high prevalence of exposure. Mov Disord 17 (suppl 5):S80.

Wang SJ, Fuh JL, Liu CY, Lin KP, Chang R, Yih JS, Chou P, Lin K, Teng EL, Larson EB, Graves AB, Liu HC. 1994a. Parkinson's disease in Kin-Hu, Kinmen: A community survey by neurologists. Neuroepidemiology 13:69-74.

Wang F-L, Semchuk KM, Love EJ. 1994b. Reliability of environmental and occupational exposure data provided by surrogate respondents in a case-control study of Parkinson's disease. J Clin Epidemiol 47:797-807

Wechsler LS, Checkoway H, Franklin GM, Costa LG. 1991. A pilot study of occupational and environmental risk factors for Parkinson's disease. Neurotoxicology 12:387-392.

Werneck AL, Alvarenga H. 1999. Genetics, drugs and environmental factors in Parkinson's disease. A case-control study. Arq Neuropsiquiatr 57:347-355.

Widdowson PS, Farnworth MJ, Simpson MG, Lock EA. 1996. Influence of age on the passage of paraquat through the blood-brain barrier in rats: A distribution and pathological examination. Hum Exp Toxicol 15:231-236.

Wong GF, Gray CS, Hassanein RS, Koller WC. 1991. Environmental risk factors in siblings with Parkinson's disease. Arch Neurol 48:287-289.

Woolley DE, Gietzen DW, Gee SJ, Magdalou J, Hammock BD. 1989. Does paraquat (PQ) mimic MPP^+ toxicity? Proc West Pharamcol Soc 32:191-193.

Yesalis III CE, Lemke JH, Wallace RB, Kohout FJ, Morris MC. 1985. Health status of the rural elderly according to farm work history: The Iowa 65+ rural health study. Arch Environ Health 40:245-253.

Zayed J, Ducic S, Campanella G, Panisset JC, André P, Masson H, Roy M. 1990. Environmental factors in the etiology of Parkinson's disease. Can J Neurol Sci 17:286-291.

Zhang J, Fitsanakis VA, Gu G, Jing D, Ao M, Amarnath V, Montine TJ. 2003. Manganese ethylene-bis-dithiocarbamate and selective dopaminergic neurodegeneration in rat: A link through mitochondrial dysfunction. J Neurochem 84:336-346.

Zorzon M, Capus L, Pellegrino A, Cazzato G, Zivadinov R. 2002. Familial and environmental risk factors in Parkinson's disease: A case-control study in north-east Italy. Acta Neurol Scand 105:77-82.

Table S1 Summary of case-control studies investigating the risk of Parkinson's disease and pesticide exposure

Reference	Cases				Controls			
	Source	Definition	Number	Age (years)	Source	Definition	Number	Matching
Ho et al. (1989), Hong Kong	Geriatric day hospital and old peoples home	Cardinal signs[a], exclusion criteria applied	35	Range: 65–87	As cases	No disease specified	105	Age, sex and source of case
Tanner et al. (1989), China	Neurology clinic	≥ 2 Cardinal signs, exclusion criteria applied	100	Mean: 57.2 Range: 31–77	As cases	Non-PD diagnosis	200	Age and sex
Golbe et al. (1990), USA	Movement disorder clinic	Not reported, but exclusion criteria were applied	106	Not reported	Cases	Spouse	106	None
Hertzman et al. (1990), Canada	Physician lists	Generally accepted criteria	57	Range: 50–79	Regional electoral roll	None	122	Age and sex
Koller et al. (1990), USA	Movement disorder clinic	≥ 2 Cardinal signs, exclusion criteria applied	150	Mean: 66	Neurology and medical clinics	Excluded if signs of parkinsonism, exposure to neuroleptics, or severe dementia	150	Age and sex
Zayed et al. (1990), Canada	GPs and neurology consultants	≥ 3 Cardinal signs, exclusion criteria	42	Mean: 57.8 SD: 11.1	Telephone directory	PD excluded	84	Age and sex

		applied						
Stern et al. (1991), USA	Neurology clinic	Not reported, but exclusion criteria were applied	149	YOPD <40 OOPD >60	Case nominations or hospital patient/volunteer	Non-parkinsonism	149	Age, sex and race
Wechsler et al. (1991), USA	Neurology clinic and PD support groups	Not reported	34	Mean: 68.4	Neurology clinic	Non-parkinsonism	25	Yes – no details given
Wong et al. (1991), USA	Hospital PD centre	≥ 2 Cardinal signs, exclusion criteria applied	38	Mean: 68.4	Neurology and general medicine clinics	Non-PD diagnosis	38	Yes – no details given
Falope et al. (1992), Nigeria & USA	Neurology clinic	≥ 3 Cardinal signs	50	Mean: 65.9	Medical clinics	Non-PD diagnosis	50	Age and sex
Jiménez-Jiménez et al. (1992), Spain	Movement disorder clinic	Not reported	128	Mean: 66.8 SD: 9.1	Hospital emergency room	Non-neurological ailments	256	Age and sex
Semchuk et al. (1992), Canada	Hospital case register	≥ 2 Cardinal signs	130	Mean: 68.5 SD: 11.5	Community	Non-parkinsonism and no dementia	260	Age and sex
Butterfield et al. (1993), USA	YOPD support groups	≥ 2 Cardinal signs, exclusion criteria applied	63	All <50	Catchment area of cases	Diagnosed with rheumatoid arthritis	68	Frequency matched for age sex, year of birth and diagnosis
Hubble et al. (1993), USA	Rural: PD outreach clinic	≥ 2 Cardinal signs, exclusion criteria	31	Mean: 69 SD: 10.2	Rural: news media and senior citizens'	Signs of PD or other neurodegenerative	45	Not reported

		applied			lunch program	disease excluded		
	Urban: PD clinic and PD support groups		32		Urban: neurology and case contacts		31	
Hertzman et al. (1994), Canada	GPs and hospital records	≥ 2 Cardinal signs, exclusion criteria applied	127	Mean: 70.5	Electoral rolls and hospital records	Healthy subjects and community cardiac disease patients	245	No
Morano et al. (1994), Spain	Neurology clinic	Hoehn and Yahr staging criteria	74	Mean: 65.4 SD: 1.1	Accident and Emergency, and Neurology outpatients	Non-neurological and functional CNS pathology	148	Not reported
Chaturvedi et al. (1995), Canada	Cohort of Canadians ≥ 65 years	Not reported	87	≥ 65	As cases	Not reported	2070	Not reported
Seidler et al. (1996), Germany	Neurology clinics	UK PD Brain Bank criteria	380	Mean: 52.5 SD: 6.6	Random addresses	Non PD	755	Age and sex
Liou et al. (1997), Taiwan	Movement disorder clinic	≥ 2 Cardinal signs	120	Mean: 58.3	Outpatient clinics	Non-PD diagnoses, previous brain disease excluded	240	Yes
Chan et al. (1998), Hong Kong	Neurology outpatients clinic	≥2 Cardinal signs, plus others and family history	215	<60–80+	Hospital	No disease reported	313	Yes
Gorell et al. (1998), USA	Health care provider	Not reported, but exclusion criteria	144	>50	As cases	No disease	464	Frequency matched for

		were applied						age and sex
McCann et al. (1998), Australia	Hospital, residential care home and community groups	≥ 2 Cardinal signs	224	Mean: 70.3<br>SD: 0.6	As cases	No disease	310	Yes
Menegon et al. (1998), Australia	Community groups, hospital wards and outpatient clinics	Cardinal signs, exclusion criteria applied	95	Mean: 72<br>SD: 9	As cases	Healthy	95	Not reported
Smargiassi et al. (1998), Italy	Hospital outpatients	UK PD Brain Bank criteria	86	Mean: 66.4<br>SD: 9.7	As cases	Cardiology, nephrology, ophthalmology, and dermatology patients	86	Not reported
Fall et al. (1999), Sweden	GP lists and prescription records	≥ 1 Cardinal sign and progression, exclusion criteria applied	113	Mean: 62.9<br>SD: 8.9	Regional population register	No disease	263	Not reported
Kuopio et al. (1999a), Finland	Community	UK PD Brain Bank criteria and no dementia	123	Mean: 68.7<br>SD: 8.9	Population register	Healthy	246	Age, sex and municipality
Taylor et al. (1999), USA	Movement disorder clinic	≥ 2 Cardinal signs, L-dopa responsive, and other signs	140	Range: 31–88	In-laws and friends nominated by cases	Healthy	147	Yes
Werneck and Alvarenga	Neurology clinic	UK PD Brain Bank	92	Mean: 70.6	Neurology clinic	Non-parkinsonism	110	Age and sex

(1999), Brazil		criteria						
Nelson et al. (2000), USA[b]	Medical care program	Not reported	496	Not reported	As cases	Healthy	541	Frequency matched for age and sex
Preux et al. (2000), France	Inpatient and outpatients	UK PD Society criteria	140	Mean: 71.1 SD: 7.5	As cases	Ophthalmology, ENT, endocrinology and rheumatology patients	280	Age and sex
Behari et al. (2001), India	Movement disorder clinic	≥ 2 Cardinal signs, progression, and responsive to L-dopa	377	Mean: 56.8 SD: 11.1	Neurology clinic	Non-parkinsonism	377	Age and sex
Herishanu et al. (2001), Israel	Neurology clinic	≥ 2 Cardinal signs, progression, and responsive to L-dopa	93	Not reported	Hospital neurology dermatology, internal medicine, and outpatient clinics	Non-PD diagnosis, those with extrapyramidal signs or neurodegenerative disease excluded	93	Age and sex
Kamel et al. (2001), USA[b]	Cohort of licensed pesticide applicators	Self-reported PD	55	Not reported	As cases	Non-PD	22 286	Not reported
Kirkey et al. (2001), USA	Health care provider	Not reported	144	>50	As cases	Disease free	464	Frequency matched for age and sex
Vidal et al. (2002), France[b]	Insurance service	Neurologist	227	Not reported	As cases	PD free	562	Age, sex and

		confirmed						place of residence
Zorzon et al. (2002), Italy	PD Centre and movement disorder clinic	≥ 2 Cardinal signs, asymmetry and clinical progression, exclusion criteria applied	136	Mean: 70 SD: 9.2	Neurology clinic	Non-parkinsonism	272	Age and sex
Duzcan et al. (2003), Turkey	Village	≥ 2 Cardinal signs or advice of relatives of deceased	36	>50	As cases	Non-neurological	108	Age and sex
Firestone et al. (2005), USA	Health care provider	≥2 Cardinal signs, one of which had to be bradykinesia or resting tremor	250	Range 37–88	As cases	Non-neurological	388	Age, sex and source of case

Abbreviations: CNS, central nervous system; ENT, ear, nose and throat; GP, General Practioner; OOPD, old onset Parkinson's disease; PD, Parkinson's disease; SD, Standard deviation; YOPD, young onset Parkinson's disease

[a]Cardinal signs: tremor, rigidity, bradykinesia, postural instability. [b]Reported as conference abstracts.
